# Single-ascending and multiple-ascending dose study of the pharmacokinetics, safety, and tolerability of BV100 (rifabutin for infusion) in healthy volunteers

**DOI:** 10.1128/aac.01582-25

**Published:** 2026-03-02

**Authors:** Christian Kemmer, Martin Hirsch, Christian Reh, Myriam Davila, Françoise Jung, Lisa Husband, Glenn E. Dale

**Affiliations:** 1 BioVersys AG 727754 , Basel, Switzerland; 2 Nuvisan GmbH 205453 , Neu-Ulm, Bavaria, Germany; 3 TFS 651324 , Barcelona, Spain; 4 BioVersys SAS, Lille, France; Providence Portland Medical Center, Portland, Oregon, USA

**Keywords:** BV100, rifabutin, pharmacokinetics

## Abstract

BV100 (rifabutin for infusion) is being developed as an intravenous formulation for treating serious or life-threatening infections due to carbapenem-resistant *Acinetobacter baumannii* in patients with limited treatment options. Phase 1 studies were conducted to characterize the pharmacokinetics (PK), safety, and tolerability of BV100 in healthy volunteers after single and multiple doses. Single-ascending and multiple-ascending dose studies were conducted in healthy subjects to establish the PK profile of BV100. Blood samples were assayed to determine plasma concentrations of rifabutin and the major metabolite 25-O-desacetyl-rifabutin and to determine PK parameters. Subjects were assessed for safety and tolerability. The PK profile of BV100 was generally dose-proportional in the single-ascending dose studies with a *t*_1/2_ of 7.9–56.1 h, and *T*_max_ of 1.0–1.75 h and increasing exposure with dose. A q12h versus q24h dosing interval resulted in approximately a 1.5-fold to 2-fold greater exposure of rifabutin. The 25-O-desacetyl-rifabutin metabolite represented <5% of rifabutin activity. Adverse events were more common at higher doses with q24h versus q12h dosing, and with a 60 min infusion time. The most common adverse events were infusion site events, with systemic treatment-emergent adverse events as expected for the known safety profile of rifabutin. No other treatment-related safety issues were identified. BV100 demonstrated a dose-proportional PK profile and was generally safe and well tolerated. Based on these results, BV100 doses of 200–300 mg q12h are undergoing evaluation in a Phase 2 study for treating carbapenem-resistant *A. baumannii* infections.

**CLINICAL TRIALS:**

This study is registered with ClinicalTrials.gov as NCT04636983 and NCT05087069.

## INTRODUCTION

Antimicrobial resistance is a growing problem worldwide, and carbapenem-resistant *Acinetobacter baumannii* (CRAB), multidrug-resistant *Pseudomonas aeruginosa*, and carbapenem-resistant and extended-spectrum beta-lactamase-producing Enterobacterales have been identified as urgent or serious threats by the Centers for Disease Control and Prevention and World Health Organization (1, 2).

*Acinetobacter baumannii* is one of the most important and common pathogens causing nosocomial outbreaks worldwide, especially in intensive care units. The most common bodily site of *A. baumannii* infection is the respiratory tract, particularly in cases of hospital-acquired bacterial pneumonia (HABP) (3). Today, a substantial proportion of these isolates are CRAB. Carbapenem resistance rates exceed 90% in some parts of the world, and mortality rates for the most common CRAB infections, i.e., nosocomial pneumonia and bloodstream infections (BSIs), may approach 60%. More recently, the rise in the frequency of nosocomial infections caused by extensively drug-resistant (XDR) or pan drug-resistant (PDR) *A. baumannii* strains has been of great concern because XDR/PDR resistance has been associated with high mortality and treatment failure (4, 5). Currently, *A. baumannii* is recognized as one of the most difficult healthcare-associated infections to control and treat, and the optimal treatment of infections caused by XDR *A. baumannii* has not been established (6, 7). In the latest reports on the antibiotic-resistance threats in the United States, the CDC escalated the threat level of CRAB to urgent, indicating the lack of treatment options for these infections (1, 8).

Treatment for infections due to *A. baumannii* generally consists of carbapenems, third-generation cephalosporins, glycylcyclines, or fluoroquinolones with or without aminoglycosides, with a typical treatment duration of 7–14 days (9); however, there are difficulties in establishing the effectiveness of treatment regimens as there is no clear standard of care.

The antimicrobial activity of the rifamycins is based on their ability to penetrate the bacterial cell wall and inhibit DNA-dependent RNA polymerase, leading to inhibition of the RNA transcription and subsequent bacterial protein synthesis. The binding constants for prokaryotic RNA polymerases are 10,000-fold higher than those for eukaryotic enzymes. The significantly higher penetration through the bacteria cell wall of gram-positive bacteria accounts for the higher activity against gram-positive pathogens when compared to gram-negative organisms, despite their similar RNA polymerase inhibitory activities (10). Rifabutin is a spiro-piperidyl-rifamycin derived from rifamycin-S and belonging to the class of ansamycins (11). To exert its antibacterial activity, rifabutin must penetrate the bacterial cell membrane in order to bind to its target enzyme (12). In an iron-depleted growth medium, the *in vitro* activity of rifabutin against *A. baumannii* was found to be increased 200-fold (13). However, the low peak plasma concentrations of rifabutin after oral dosing and a highly variable bioavailability limit its utility for the treatment of serious infections (14).

BV100 (rifabutin for infusion) is being developed to achieve clinically meaningful exposures and to prevent rapid resistance development. The new formulation uses dimethyl isosorbide (DMI) to significantly enhance systemic rifabutin exposures following intravenous (IV) administration of BV100. *In vitro*, rifabutin has potent bactericidal activity against *A. baumannii* including carbapenem-resistant, XDR, and PDR isolates with a minimal inhibitory concentration (MIC_90_) of 1 mg/L (15). This activity was confirmed in animal models of *A. baumannii* infection, including lung and bloodstream infections (16–18). *In vivo*, BV100 demonstrated synergy with polymyxins against *A. baumannii* (19). The pharmacodynamic (PD) index for BV100 that best correlated with efficacy in an *A. baumannii* neutropenic mouse lung infection model was the ratio of the area under the unbound drug concentration-time curve to the MIC (*f*AUC/MIC), with a lower dependence on the ratio of the maximal unbound (free) drug concentration to the MIC (*fC*_max_/MIC) (17).

BV100 is being developed for the treatment of serious or life-threatening infections due to CRAB in patients with limited treatment options, including ventilator-associated bacterial pneumonia, HABP, and BSI. Phase 1 studies were conducted to characterize the pharmacokinetics (PK), safety, and tolerability of BV100 in healthy volunteers after single and multiple doses.

## MATERIALS AND METHODS

All studies were conducted in accordance with the principles of the Declaration of Helsinki (20), European Union Directives (21–23), local guidelines (24), and Good Clinical Practices (25).

### Study design

#### Single-ascending dose

This was a Phase 1, double-blind, randomized, placebo-controlled study of single-ascending doses of IV BV100 in healthy male subjects to investigate the safety, tolerability, and pharmacokinetics (clinicaltrials.gov NCT04636983). A screening period occurred from Day −21 to Day −2 to determine study eligibility. Subjects were hospitalized from Day −1 until discharge on Day 5. On Day 6, subjects returned for an ambulatory visit, and follow-up assessments were performed between Day 7 and Day 10. The total time in the study was up to 31 days. Subjects were randomized in a 2:6 ratio in sequential dose groups of placebo (physiological saline) or BV100 at doses of 0.5, 1.5, 3.0, 6.0, or 9 mg/kg infused IV over 60 min. Additional dose groups were 9 mg/kg infused over 120 min and two doses of 450 mg infused over 120 min at a 12 h interval. For each dose group, two sentinel subjects (one active and one placebo) were dosed first. This was followed by a blinded safety data review up to 48 h post-dose by the investigator and the sponsor before dosing the remaining subjects. Upon completion of each dose group and prior to dose escalation or proceeding with the next cohort with alternative infusion times, a Safety Monitoring Committee (SMC) reviewed and analyzed available safety and preliminary PK data until Day 5 from at least six subjects from the previous dose group to decide whether to continue with the next cohort.

#### Multiple-ascending dose

This was a Phase 1, double-blind, randomized, placebo-controlled, multiple-ascending dose study to investigate the safety, tolerability, and pharmacokinetics of BV100 as multiple IV doses to healthy male subjects (clinicaltrials.gov NCT05087069). A screening period occurred from Day −21 to Day −2. Eligible subjects were hospitalized throughout the treatment period from Day −1 until discharge on Day 11. Follow-up assessments were performed on Day 12 (phone call) and between Day 18 and Day 21 to assess safety and tolerability as an outpatient. The total time in the study was up to 43 days, the hospitalization phase (12 days), one phone call, and the follow-up visit. Subjects were randomized in a 2:6 ratio to receive placebo (physiological saline) or BV100 300 mg q24h (group A) or BV100 300 mg q12h (group B) via a 120 min infusion for 7 days. Upon completion of each dose group and prior to dose escalation, the SMC reviewed and analyzed available safety and tolerability data until Day 11 from at least six subjects from the current dose group, as well as preliminary PK data until Day 11 from at least four subjects receiving BV100 from the current dose group, to decide whether to proceed with the next group.

### Subject selection

#### Single-ascending and multiple-ascending dose studies

Healthy male subjects ages 18–55 years who weighed at least 50 kg and had a body mass index (BMI) of 19–30 kg/m^2^ were eligible. Subjects were non-smokers, former smokers (<10 pack years), former users of nicotine-containing products, or stable non-smokers for at least 3 months prior to study drug administration.

### Study assessments

#### Single-ascending dose

The safety of BV100 was assessed from treatment-emergent adverse events (TEAEs), vital signs (blood pressure, heart rate, temperature, pulse oximetry, and respiratory rate), clinical laboratory testing (biochemistry, hematology, and urinalysis), physical and neurological examinations, local tolerability, serology, 12-lead electrocardiogram (ECG), and telemetry.

Whole blood samples were obtained to measure concentrations of rifabutin and 25-O-desacetyl-rifabutin in plasma. PK parameters for rifabutin included AUC_0-12_, AUC_0-24_, AUC_0-96_, AUC_0-tlast_, AUC_0-inf_, *C*_max_, *T*_max_, *t*_1/2_, CL, *V*_*z*_, *V*_ss_, and MRT. For 25-O-desacetyl-rifabutin, PK parameters included AUC_0-12_, AUC_0-24_, AUC_0-tlast_, *C*_max_, and *T*_max_. Blood samples were collected pre-dose, 5, 30, 60, 70, and 90 min, and 2, 2 h 5 min, 2 h 10 min, 2.5, 3, 4, 5, 6, 8, 12, 24, 48, 72, 96, 120 h after the start of the infusion. For the 450 mg q12h dose group, blood samples were collected pre-dose, 5, 30, 60, 70, and 90 min, and 2, 2 h 5 min, 2 h 10 min, 2.5, 3, 4, 5, 6, 8, and 12 h after the start of the infusion. The method validation and analysis of the study samples were performed in compliance with the Standard Operating Procedures based on the European Medicines Agency (EMA) and FDA Guidelines on Bioanalytical Method Validation and the Reflection Paper for Laboratories that Perform the Analysis or Evaluation of Clinical Trial Samples. Urine samples were collected for 96 h after the start of infusion at time intervals of 0–4, 4–8, 8–12, 12–24, 24–48, 48–72, and 72–96 h to determine CL_R_, Ae_0-24_, and Ae_0-96_.

#### Multiple-ascending dose

Safety was evaluated from physical and neurological examinations, vital signs (blood pressure, heart rate, temperature, pulse oximetry, and respiratory rate), 12-lead ECG, local tolerability with the Visual Inspection Phlebitis score (26), serology, clinical laboratory testing (chemistry, hematology, and urinalysis), and TEAEs.

Whole blood samples were obtained to measure concentrations of rifabutin and 25-O-desacetyl-rifabutin in plasma. Blood samples were obtained prior to the start of each infusion on Days 1–7 and every 24 h until 96 h after the last dose on Days 8, 9, 10, and 11. On Days 1 and 7, blood samples were obtained pre-dose and at 5, 30, 60, 70, 90, 120, 125, and 130 min, and 2.5, 3, 4, 5, 6, 8, and 12 h post-dose. Urine was collected from 0 to 24 h after the start of infusion on Day 7. For Day 1, PK parameters for rifabutin included AUC_0-12_, AUC_0-24_, *C*_max_, and *T*_max_. At Day 7, PK parameters were AUC_0-12_, AUC_0-24_, AUC_0-96_, AUC_0-tlast_, AUC_0-inf_, *C*_max_, *C*_av_, *T*_max_, *t*_1/2_, λz, CL, and *V*_*z*_. Plasma concentrations of 25-O-desacetyl-rifabutin were used to determine AUC_0-12_, AUC_0-24_, AUC_0-tlast_ (Day 7 only), *C*_max_, and *T*_max_. On Day 7, CL_R_ and Ae_0-24_ of rifabutin in urine were determined.

### Bioanalytic methods

Plasma and urine concentrations of rifabutin and 25-O-desacetyl-rifabutin were determined using a validated liquid chromatography with tandem mass spectrometry method (27). The limit of quantification (LOQ) for plasma concentrations of rifabutin and 25-O-desacetyl-rifabutin in plasma was 20 ng/mL and 5 ng/mL, respectively. The LOQ for rifabutin in urine was 5 ng/mL.

### Statistical analysis

Descriptive and inferential analyses were performed using SAS, version 9.4. Non-compartmental PK analysis was performed using Phoenix WinNonlin, version 7.0 or higher.

#### Single-ascending dose

For PK parameters, geometric mean, geometric standard deviation (SD), and geometric coefficient of variation (CV) were derived. To explore PK dose proportionality for AUC_0-24_, AUC_0-tlast_, and AUC_0-inf_, a power model was used. PK parameters and dose values were logarithmically transformed prior to analysis and evaluated with an analysis of variance (ANOVA) including log(dose) as a fixed effect. Linear-dose proportionality was concluded if the two-sided 90% confidence interval (CI) for the slope value of log-transformed dose was within the critical region of 0.5–2.

#### Multiple-ascending dose

For PK parameters, geometric mean, geometric SD, and geometric CV were derived. To explore the PK dose proportionality of rifabutin on AUC_0-24_, AUC_0-inf_, and *C*_max_ on Day 7, a power model was used. PK parameters and dose value were logarithmically transformed prior to analysis and evaluated using an ANOVA including log(dose) as a fixed effect. The linear-dose proportionality was concluded in an exploratory manner if the two-sided 90% CI for the slope value of log transformed dose was within the critical region {[1 + ln(LL)/ln(*r*)]; [1 + ln(UL)/ln(*r*)]}, where *r* = dose maximum/dose minimum, LL = 0.5, and UL = 2.0.

## RESULTS

### Single-ascending dose

Fifty-three subjects were randomized and included in the safety analysis. Of 39 subjects randomized to BV100, 1 discontinued for a TEAE and was excluded from the PK analysis. All subjects were male and white, and one subject was Hispanic or Latino. Mean age was 37.0 years (range: 18–55 years), mean weight was 78.8 kg (range: 62.4–106.3 kg), mean BMI was 25.2 kg/m^2^ (range: 19.7–30.0 kg/m^2^), and mean eGFR was 108.8 mL/min/1.73 m^2^ (range: 80–144 mL/min/1.73 m^2^) (Table 1).

**TABLE 1:** Baseline characteristics for single-ascending dose study of BV100^*a*^

Parameter	Placebo (*N* = 14)	0.5 mg/kg (*N* = 6)	1.5 mg/kg (*N* = 6)	3 mg/kg (*N* = 6)	6 mg/kg (*N* = 6)	9 mg/kg (*N* = 5)	9 mg/kg (*N* = 4)	450 mg q12h (*N* = 6)
Age, years	36.1 ± 13.3	41.0 ± 11.2	41.3 ± 10.8	37.5 ± 13.7	45.3 ± 9.2	35.8 ± 8.8	30.3 ± 9.8	27.8 ± 10.0
Age range, years	19–55	25–53	25–53	25–55	31–54	28–51	21–44	18–46
Weight, kg	78 ± 12	73 ± 6	78 ± 9	80 ± 7	83 ± 12	80 ± 11	83 ± 4	77 ± 8
BMI, kg/m^2^	25.7 ± 2.9	24.5 ± 2.5	25.2 ± 2.5	24.9 ± 1.7	25.7 ± 2.6	24.5 ± 3.0	26.0 ± 1.8	24.2 ± 1.7
eGFR, mL/min/1.73 m^2^	109 ± 16	105 ± 14	130 ± 12	108 ± 15	93 ± 9	101 ± 10	108 ± 10	116 ± 8

^*a*^ Values are mean ± standard deviation. BMI, body mass index; eGFR, estimated glomerular filtration rate.

#### Pharmacokinetics

**Fig 1:**
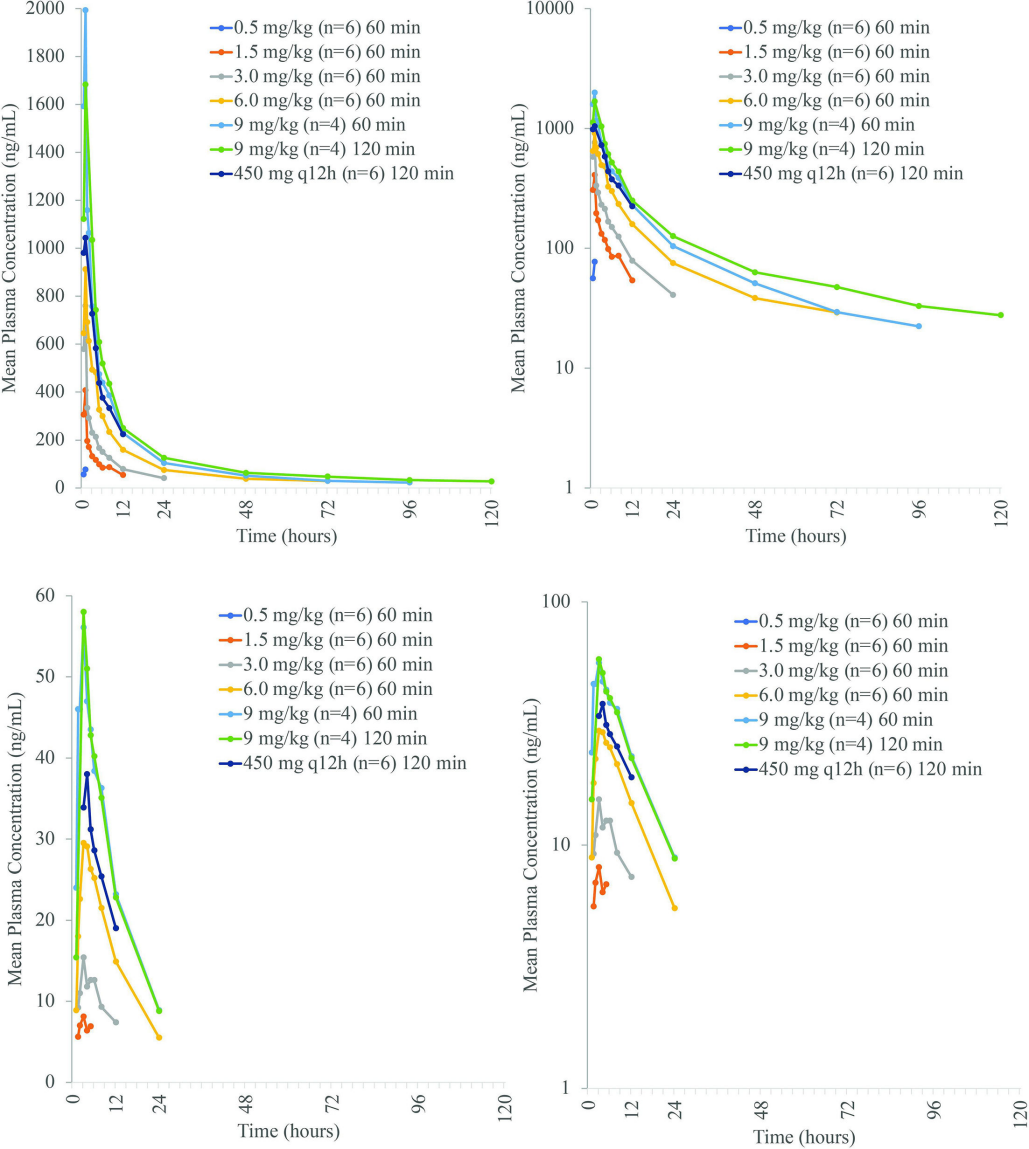
Linear (left) and semi-log (right) arithmetic mean plasma rifabutin (top) and 25-O-desacetyl-rifabutin (bottom), plasma concentrations following single-ascending doses of BV100 in healthy subjects (PK population).

Following a single dose of BV100, mean plasma rifabutin PK parameters were generally dose-proportional. Peak rifabutin concentrations were reached at approximately 1 h (end of infusion) with a 60 min infusion (Fig. 1). With 120 min infusion times, peak concentrations were reached at 1.75 h with 9.0 mg/kg and at 1.5 h with 450 mg q12h (Table 2). Half-life ranged from 7.9 h at 0.5 mg/kg to 54.1 h with 6.0 mg/kg. Mean half-life was markedly lower (37.8 h) when 9.0 mg/kg was infused over 60 min compared to 120 min (56.1 h). The decision to move from weight-based dosing to flat dose was based on the population model of oral rifabutin, in which weight, height, and body surface area did not have an influence on pharmacokinetic parameters (28).

**TABLE 2:** Plasma PK parameters for rifabutin after single-ascending doses of BV100^*c*^

	60 min infusion					120 min infusion	
Parameter	0.5 mg/kg (*N* = 6)	1.5 mg/kg (*N* = 6)	3 mg/kg (*N* = 6)	6 mg/kg (*N* = 6)	9 mg/kg (*N* = 4)	9 mg/kg (*N* = 4)	450 mg q12h (*N* = 6)
AUC_0-12_, h·ng/mL	NA	NA	NA	NA	NA	NA	6,268 (23.9)
AUC_0-24_, h·ng/mL^*a*^	572 (33.7)	1,840 (26.0)	3,087 (15.5)	5,733 (15.9)	9,242 (21.7)	11,080 (14.5)	NA
AUC_0-96_, h·ng/mL^*a*^	644 (35.3)	2,297 (45.0)	4,230 (26.8)	8,504 (16.3)	12,640 (23.9)	15,593 (16.6)	NA
AUC_0-120_, h·ng/mL^*a*^	645 (35.3)	2,337 (49.3)	4,325 (29.1)	8,979 (17.6)	13,070 (25.3)	16,329 (18.2)	NA
AUC_0-tlast_, h·ng/mL	185 (182.1)	1,926 (63.7)	3,756 (32.4)	8,535 (21.6)	12,683 (28.9)	16,130 (20.4)	NA
AUC_0-inf_, h·ng/mL^*b*^	–^*d*^	2,210 (14.7)	4,073 (26.7)	9,735 (26.6)	14,170 (29.6)	17,468 (24.9)	NA
*C*_max_, ng/mL	76 (41.0)	400 (25.7)	743 (36.9)	905 (24.8)	1,677 (83.1)	2,265 (13.3)	1,414 (43.9)
CL, mL/h/kg^*b*^	–	679 (14.7)	737 (26.7)	616 (26.6)	635 (29.6)	515 (24.9)	NA
*V*_*z*_, mL/kg^*b*^	–	9,386 (20.6)	16,437 (19.8)	41,675 (12.0)	34,620 (46.3)	37,473 (43.1)	NA
*V*_ss_, mL/kg^*b*^	–	7,747 (20.0)	122,017 (16.1)	26,267 (12.6)	19,712 (29.9)	19,521 (48.0)	NA
MRT, h^*b*^	–	11.4 (13.8)	16.3 (35.1)	42.6 (36.2)	31.0 (50.9)	37.9 (74.9)	NA
*T*_max_, h	1.0 (0.5–1.0)	1.0 (0.5–1.0)	1.0 (0.5–1.0)	1.0 (1.0–2.0)	1.25 (1.0–2.0)	1.75 (1.5–2.0)	1.5 (0.5–2.0)
*t*_1/2_, h	7.9 (14.1)	13.0 (110.6)	19.6 (79.4)	54.1 (47.5)	37.8 (73.9)	56.1 (62.1)	NA
λ_z_, l/h	0.09 (14.1)	0.05 (110.6)	0.04 (79.4)	0.01 (47.5)	0.02 (73.9)	0.01 (62.1)	NA

^*a*^ *n* = 4 for 0.5 mg/kg, 60 min, *n* = 6 for 1.5 mg/kg, 60 min, *n* = 6 for 3.0 mg/kg, 60 min, *n* = 6 for 6.0 mg/kg, 60 min,*n* = 4 for 9.0 mg/kg, 60 min, *n* = 4 for 9.0 mg/kg, 120 min.

^*b*^ n = 0 for 0.5 mg/kg, 60 min, *n *= 3 for 1.5 mg/kg, 60 min, *n *= 5 for 3.0 mg/kg, 60 min; *n* = 4 for 6.0 mg/kg, 60 min, *n* = 4 for 9.0 mg/kg, 60 min; *n* = 3 for 9.0 mg/kg, 120 min.

^*c*^ Values are geometric mean (% coefficient of variation) except for *T*_max_ which is median (range). NA, not applicable.

^*d*^ –, not calculated (n = 0).

**Fig 2:**
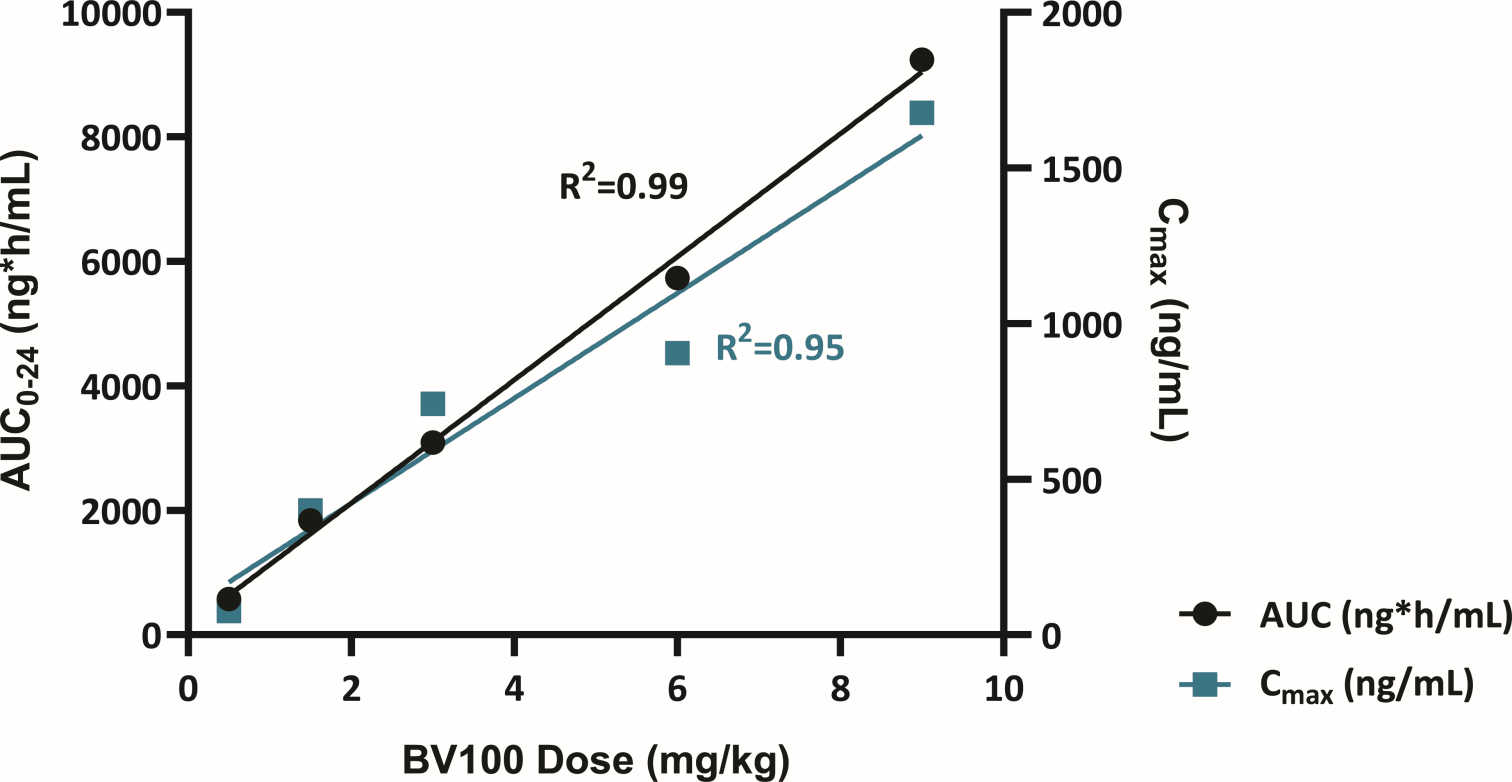
Rifabutin AUC and *C*_max_ increase linearly with dose.

For AUC_0-24_, the point estimate for the slope of the regression line was 0.927 (90% CI: 0.850–1.004). For AUC_0-inf_, the point estimate for the slope of the regression line was 1.073 (90% CI: 0.938–1.207). Both CIs were contained within the pre-defined critical region [0.76; 1.24]. Therefore, dose proportionality was assumed for the dose range of 0.5–9.0 mg/kg infused over 60 min (Fig. 2). For AUC_0-tlast_, the point estimate for the slope of the regression line was 1.448 (90% CI: 1.229–1.666). As the CI exceeded the pre-defined critical region [0.76; 1.24], the increase was more than dose-proportional in the dose range of 0.5–9.0 mg/kg. Mean cumulative urinary excretion of rifabutin generally increased with doses from 3.0 to 9.0 mg/kg (Table S1). In contrast, renal clearance decreased as doses increased. For 25-O-desacetyl-rifabutin, exposure (AUC_0-24_, AUC_0-tlast_, and *C*_max_) generally increased with dose and represented approximately 5% of parent rifabutin concentrations (Table S2; Fig. 1). *T*_max_ with a 60 min infusion was 2.5–3.0 h compared with 1.0 h for rifabutin.

### Multiple-ascending dose

Sixteen patients were randomized and dosed, and 14 completed the study. Two subjects discontinued the study for TEAEs and were not included in the PK analysis. Mean age was 36.5 years (range: 23–55 years), mean weight was 78.6 kg (range: 63.7–91.4 kg), mean BMI was 24.1 kg/m^2^ (range: 20.3–28.1 kg/m^2^), and mean GFR was 114.7 mL/min/1.73 m^2^ (range: 77–135 mL/min/1.73 m^2^). All subjects were male, and 15 (93.8%) were white, with 1 black subject.

#### Pharmacokinetics

**Fig 3:**
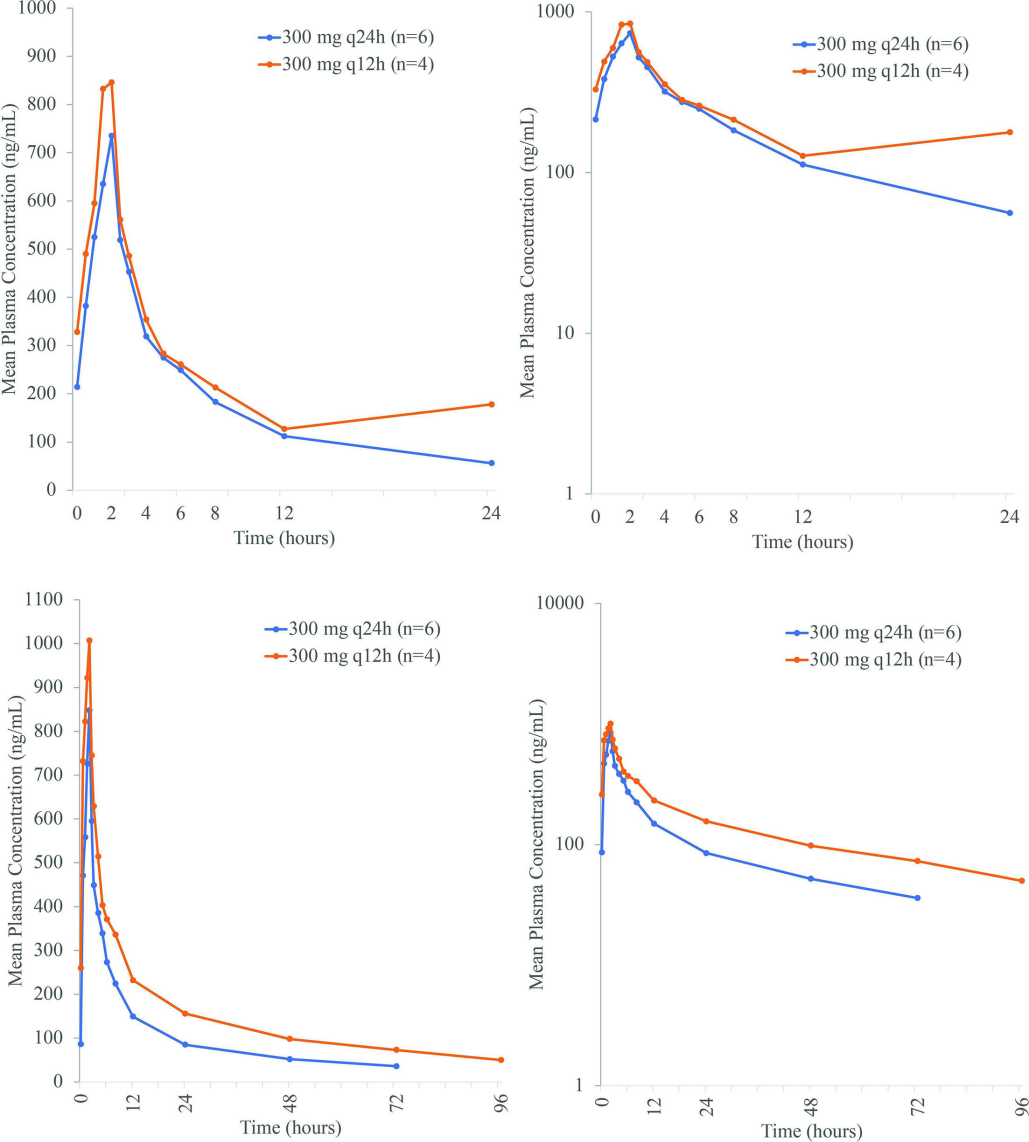
Linear (left) and semi-log (right) arithmetic mean plasma concentrations of rifabutin at Day 1 (top) and Day 7 (bottom) after BV100 300 mg q24h and 300 mg q12h in the MAD study (PK population).

**Fig 4:**
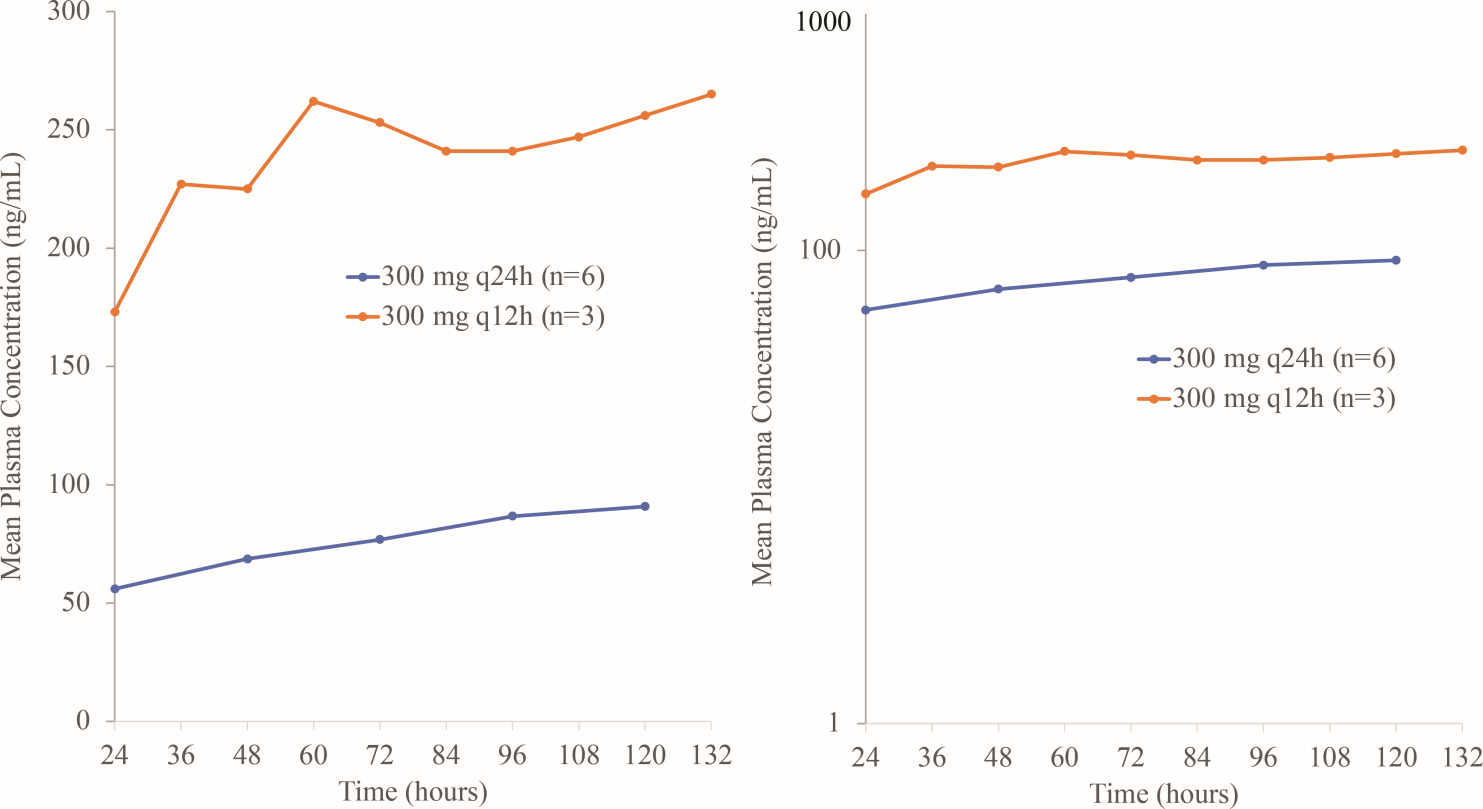
Linear (left) and semi-log (right) arithmetic mean trough plasma concentrations of rifabutin for 300 mg q24h and 300 mg q12h from Day 2 to Day 6 (PK analysis without subjects with contaminated values; *N* = 9).

Peak rifabutin concentrations were reached 2 h after starting the infusion on Day 1 and Day 7 (Fig. 3). On Day 1, maximum geometric mean concentrations were 721.6 ng/mL with 300 mg q24h and 894.2 ng/mL with 300 mg q12h (Table 3). On Day 7, mean pre-dose rifabutin plasma concentrations were approximately threefold higher with 300 mg q12h (260.3 ng/mL) than with 300 mg q24h (85.7 ng/mL). On Day 1, the geometric mean AUC_0-12_ of rifabutin was similar with 300 mg q24h and 300 mg q12h (Table 3). However, on Day 7, the geometric mean AUC_0-24_ of rifabutin was 1.5-fold higher for 300 mg q12h compared with 300 mg q24h, the geometric mean AUC_0-tlast_ was 1.7-fold higher with 300 mg q12h compared with 300 mg q24h, and the geometric mean AUC_0-inf_ was 2.0-fold higher for 300 mg q12h compared with 300 mg q24h. For 300 mg q24h, arithmetic mean trough concentrations showed a slight and steady increase, which stabilized from 96 h onward. In contrast, arithmetic mean trough concentrations for 300 mg q12h increased until 60 h (262.3 ng/mL) and then were similar until 132 h (265.3 ng/mL) (Fig. 4).

**TABLE 3:** Plasma PK parameters for rifabutin after multiple-ascending doses of BV100 (PK population)^*b*^

	300 mg q24h (*n* = 6)	300 mg q12h (*n* = 4)
Day 1		
*C*_max_, ng/mL	721.6 (27.2)	894.2 (11.4)
*T*_max_, h	2.1 (2.0-2.1)	1.8 (1.5-2.0)
AUC_0-12_, h ng/mL	3,435 (18.9)	3,924 (20.1)
AUC_0-24_, h ng/mL	4,422 (19.5)	–^*c*^
Day 7		
*C*_max_, ng/mL	827.6 (24.3)	1,012.0 (15.5)
*C*_av_, ng/mL	224.4 (18.1)	466.2 (9.2)
*T*_max_, h	2.0 (2.0, 2.0)	2.0 (2.0, 2.2)
AUC_0-12_, h ng/mL	3,995 (18.6)	5,594 (9.2)
AUC_0-24_, h ng/mL	5,385 (18.1)	7,916 (8.7)
AUC_0-96_, h ng/mL	8,810 (20.6)	14,342 (18.3)
AUC_0-tlast_, h ng/mL	8,629 (22.2)	14,343 (18.3)
AUC_0-inf_, h ng/mL^*a*^	8,095 (1.5)	16,413 (27.6)
CL, L/h	55.7 (18.1)	53.6 (9.2)
V_z_, L	3,630 (87.2)	3,388 (31.3)
Half-life, h	45.2 (77.3)	43.8 (26.5)
λ_z_, L/h	0.015 (77.3)	0.016 (26.5)

^*a*^ *n* = 2 for 300 mg q24h and 3 for 300 mg q12h.

^*b*^ Values are geometric mean (% coefficient of variation) except for *T*_max_, which is median (min-max).

^*c*^ –, not applicable.

Urine samples for PK assessments of rifabutin were collected on Day 7. Geometric mean cumulative urinary excretion of rifabutin until 24 h (Ae_0-24_) was 19.6 mg for 300 mg q24h and 28.4 mg for 300 mg q12h. Geometric mean renal rifabutin clearance (CL_R_) at 24 h was 3,641 mL/h with 300 mg q24h and 3,582 mL/h with 300 mg q12h. Renal clearance was more variable at the higher dose (fewer patients); thus, it is difficult to draw conclusions on dose dependency.

At Day 1, geometric mean AUC_0-12_ of 25-O-desacetyl-rifabutin was similar between 300 mg q24h (150.3 h ng/mL) and 300 mg q12h (165.5 h ng/mL) (Table S3), whereas geometric mean *C*_max_ was about 1.2-fold higher with 300 mg q12h (20.79 h ng/mL) than with 300 mg q24h (17.34 h ng/mL). At Day 7, exposure (AUC and *C*_max_) continued to be higher with 300 mg q12h than with 300 mg q24h. Comparison of Day 1 and Day 7 showed little accumulation of the metabolite. Exposure to 25-O-desacetyl-rifabutin was approximately 1.4% of rifabutin in plasma.

### Safety/tolerability

#### Single-ascending dose

Overall, 53 TEAEs were reported by 23 (43.4%) subjects (Table S4). With increasing doses, a trend was observed for a higher number of TEAEs and a higher frequency of drug-related TEAEs. No serious AEs occurred, and no subject was withdrawn from the trial. The majority of TEAEs (approximately 45%) were due to reduced local tolerability at the higher doses (≥6 mg/kg). Three (75.0%) subjects reported 9 TEAEs after BV100 9.0 mg/kg over 120 min compared with 5 (100%) subjects with 9 mg/kg over 60 min, of which 6 were related to the infusion site. In the 450 mg q12h over 120 min dose group, 4 subjects (66.7%) reported 5 TEAEs, and only 2 were related to the infusion site. Most TEAEs were of mild (45 TEAEs in 21 subjects [39.6%]) or moderate (7 TEAEs in 6 subjects [11.3%]) intensity. One severe TEAE of “pain whole arm at infusion site” occurred after 9.0 mg/kg over 60 min and was considered drug-related, leading to withdrawal of the study drug.

#### Multiple-ascending dose

Eleven (68.8%) subjects reported 84 TEAEs with BV100 (Table S4). Most TEAEs were considered drug-related; 4 TEAEs with 300 mg q24h and 5 TEAEs with 300 mg q12h were not related. The number of drug-related TEAEs was markedly higher with 300 mg q12h (58 TEAEs in 5 subjects [83.3%]) than with 300 mg q24h (17 TEAEs in 5 subjects [83.3%]). The most common drug-related TEAEs (9 subjects, 43 events) were related to the infusion site. No serious TEAEs were reported, and no subjects were withdrawn from the trial. Two subjects at 300 mg q12h discontinued treatment due to TEAEs of fever in association with clinically significant changes of hematology laboratory parameters. Of these two subjects, one had 3 TEAEs of severe intensity (lymphocyte count decreased, neutrophil count decreased, and white blood cell count decreased); all were considered drug-related and recovered/resolved.

## DISCUSSION

Results from these SAD and MAD studies in healthy male volunteers found that the PK profile of rifabutin after IV infusion of BV100 was generally dose-proportional across a range of doses up to 9 mg/kg. Administering rifabutin at a q12h interval, compared to a q24h interval, resulted in approximately 1.5-fold to 2-fold greater rifabutin exposure and a slight accumulation over time based on *C*_trough_ levels. It should be noted that 7 days of dosing may not be sufficient for true steady state, given the known PK characteristics of rifabutin when administered orally. Additionally, extending the administration time from 60 min to 120 min IV infusion enhanced rifabutin exposure and improved tolerability. The primary metabolite, 25-O-desacetyl rifabutin, represented approximately 5% of parent compound. The metabolite had comparable *in vitro* activity to rifabutin and could contribute to the total antimicrobial activity (29).

Infusion site AEs were most commonly associated with IV administration of BV100. Although it is unlikely that the excipient (DMI) used to solubilize rifabutin is responsible for the local tolerability AEs, as it has been shown to be well tolerated in toxicology studies, it cannot be excluded (30). No serious AEs occurred, and no subjects were withdrawn from the trials. BV100 was well tolerated, with systemic TEAEs consistent with the known safety profile of oral rifabutin. Increasing signs of poor local tolerability became apparent in subjects at the highest dose in the SAD study and with q12h dosing in the MAD study. This issue may be mitigated with the recommended use of a peripherally inserted central catheter (PICC) or central venous catheter in clinical practice.

With the exception of the two subjects that discontinued treatment due to AEs, including abnormal hematological parameters in the MAD study, the vast majority of laboratory parameters were within normal range in both the SAD and MAD studies with no clinically relevant time-related or dose-related changes and no relevant difference between active treatment and placebo. No safety issues were identified with ECGs, physical examinations, and vital signs.

Among carbapenem-resistant organisms, CRAB is a global threat, significantly increasing morbidity and mortality and representing a significant challenge for treatment. CRAB is designated as a critical pathogen on the priority list of antibiotic-resistant bacteria, highlighting the urgent need for the development of new antibiotics (1, 2). Carbapenem resistance among nosocomial infections caused by *A. baumannii* ranges from 50% in North America to 80% in Asia (31). The WHO and the European Center for Disease Prevention and Control report that carbapenem-resistant *Acinetobacter* spp. occurs at rates of 50% or higher (32). Treatment options for CRAB are limited, with no monotherapy or combination antibiotic regimen demonstrating clear advantages (33). Currently, there is no consensus on treatment strategies that consider both safety and efficacy due to limited data from controlled clinical trials (33–35). Treatment recommendations for CRAB vary widely but often include polymyxins in combination with other antimicrobials such as tigecycline, ampicillin-sulbactam, cefiderocol, sulbactam-durlobactam, meropenem, or fosfomycin (34, 35).

Thus, BV100 may offer a novel approach to treating serious infections due to *A. baumannii*, in particular infections with limited treatment options caused by CRAB. Based on results from these Phase 1 studies, data from *in vitro* microbiology, *in vivo* efficacy studies in the murine lung infection model, as well as extensive PK/PD modeling and probability of target attainment against CRAB, a dosage regimen of 200 or 300 mg q12h administered via a 2 h IV infusion was selected for a Phase 2 trial of BV100 combined with polymyxin B for treating patients with ventilator-associated bacterial pneumonia due to CRAB (NCT05685615).

## Data Availability

The data sets used and/or analyzed during the current study are available from the corresponding author on reasonable request.

## SUPPLEMENTAL MATERIAL

Supplemental material aac.01582-25-s0001.docx Tables S1 to S5.

ASM does not own the copyrights to Supplemental Material that may be linked to, or accessed through, an article. The authors have granted ASM a non-exclusive, world-wide license to publish the Supplemental Material files. Please contact the corresponding author directly for reuse.
