# Supplementary material for: Single-ascending and multiple-ascending dose study of the pharmacokinetics, safety, and tolerability of BV100 (rifabutin for infusion) in healthy volunteers
Source: Antimicrob Agents Chemother. 2026 Mar 2;70(4):e01582-25. doi: 10.1128/aac.01582-25 (PMC13041365; doi:10.1128/aac.01582-25)
Supplement: Supplemental material — Tables S1 to S5. [file aac.01582-25-s0001.docx]

**Supplemental Tables and Figures**

Supplemental Table 1. Urine PK parameters for rifabutin after single-ascending doses of BV100

|  | 60 minute infusion | | | | | 120 minute infusion | |
| --- | --- | --- | --- | --- | --- | --- | --- |
| Parameter | 0.5 mg/kg (N=6) | 1.5 mg/kg (N=6) | 3 mg/kg (N=6) | 6 mg/kg (N=6) | 9 mg/kg (N=4) | 9 mg/kg (N=4) | 450 mg q12h  (N=6) |
| Ae_0-24_, mg | NC | NC | 15.1 (18.3) | 23.9 (12.7) | 34.7 (11.3) | 34.8 (27.8) | NA |
| Ae_0-96_, mg | NC | NC | 21.8 (20.5) | 34.6 (20.7) | 47.7 (12.5) | 56.0 (26.8) | NA |
| CL_R_ _24_, mL/h | NC | NC | 4900 (16.5) | 4175 (13.8) | 3749 (27.3) | 3141 (40.8) | NA |
| CL_R_, mL/h | NC | NC | 5153 (20.9) | 4071 (16.4) | 3777 (29.2) | 3592 (36.4) | NA |

Values are geometric mean (% coefficient of variation)

NA = not applicable; NC = results were excluded from calculations because the storage period was superseded.

Supplemental Table 2. Plasma PK parameters for 25-O-desacetyl-rifabutin and DMI after single-ascending doses of BV100

|  | 60 minute infusion | | | | | 120 minute infusion | |
| --- | --- | --- | --- | --- | --- | --- | --- |
| Parameter | 0.5 mg/kg (N=6) | 1.5 mg/kg (N=6) | 3 mg/kg (N=6) | 6 mg/kg (N=6) | 9 mg/kg (N=4) | 9 mg/kg (N=4) | 450 mg q12h  (N=6) |
|  |  |  |  |  |  |  |  |
| AUC_0-12_, h*ng/mL | NA | NA | NA | NA | NA | NA | 291 (15.9) |
| AUC_0-24_, h*ng/mL ^a^ |  | 215 | 263 | 393 (17.3) | 614 (21.8) | 710 (8.3) | NA |
| AUC_0-tlast_, h*ng/mL ^b^ |  | 81 (134.7) | 120 (58.1) | 339 (41.0) | 648 (32.8) | 574 (68.3) | NA |
| C_max_, ng/mL ^c^ |  | 10.4 (24.4) | 15.2 (32.5) | 30.5 (25.7) | 55.4 (28.6) | 56.3 (42.0) | 39.9 (18.2) |
| T_max_, h ^c^ |  | 3.0 (2.0-8.0) | 3.0 (3.0-5.0) | 3.0 (2.0-5.0) | 2.5 (1.2-3.0) | 2.75 (2.5-5.0) | 3.5 (2.2-4.0) |

Values are geometric mean (% coefficient of variation) except for T_max_ which is median (range).

NA = not applicable

^a^ n=0 (0.5 mg/kg, 60 min); n=1 (1.5 mg/kg and 3.0 mg/kg, 60 min); n=5 (6.0 mg/kg, 60 min); n=4 (9.0 mg/kg, 60 min); n=3 (9.0 mg/kg, 120 min)

^b^ n=0 (0.5 mg/kg, 60 min); n=5 (1.5 mg/kg, 60 min); n=6 (3.0 mg/kg, and 6.0 mg/kg, 60 min); n=4 (9.0 mg/kg, 60 min and 120 min)

^c^ n=0 (0.5 mg/kg, 60 min); n=5 (1.5 mg/kg, 60 min); n=6 (3.0 mg/kg and 6.0 mg/kg, 60 min; 450.0 mg, 120 min, q12h); n=4 (9.0 mg/kg, 60 min and 120 min)

Supplemental Table 3. Plasma PK parameters for 25-O-desacetyl-rifabutin after multiple ascending doses of BV100 (PK population)

|  | 25-O-desacetyl-rifabutin | |
| --- | --- | --- |
|  | 300 mg q24h (n=6) | 300 mg q12h (n=4) |
| **Day 1** |  |  |
| C_max_, ng/mL | 17.3 (48.7) | 20.8 (31.3) |
| T_max_, h | 3.5 (3.0-6.0) | 3.0 (3.0-4.0) |
| AUC_0-12_, h*ng/mL ^a^ | 150.3 (23.7) | 165.5 (31.8) |
| AUC_0-24_, h*ng/mL ^b, d^ | 277.8 (9.6) | –– |
| **Day 7** |  |  |
| C_max_, ng/mL | 12.0 (34.9) | 17.2 (8.8) |
| T_max_, h | 3.0 (2.5-3.0) | 2.3 (2.2-3.0) |
| AUC_0-12_, h*ng/mL ^a^ | 107.9 (14.4) | 146.3 (6.7) |
| AUC_0-24_, h*ng/mL ^c^ | 204.8 (NA) | 238.5 (3.5) |
| AUC_0-tlast_, h*ng/mL | 86.1 (100.0) | 205.9 (30.2) |

Values are geometric mean (% coefficient of variation) except for T_max_, which is median (min-max).

^a^ n = 5 for 300 mg q24h and 4 for 300 mg q12h

^b^ n = 2 for 300 mg q24 h and 0 for 300 mg q12h

^c^ n = 1 for 300 mg q24 h and 3 for 300 mg q12h

^d^ n = 6 for 300 mg q24 h and 0 for 300 mg q12h

Supplemental Table 4. Treatment-emergent adverse events (TEAEs) after single ascending doses of BV100 (safety population)

|  | Number (%) of Subjects/Events | | | | | | | |
| --- | --- | --- | --- | --- | --- | --- | --- | --- |
| Parameter | Placebo (N=14) | 0.5 mg/kg (N=6) | 1.5 mg/kg (N=6) | 3 mg/kg (N=6) | 6 mg/kg (N=6) | 9 mg/kg (N=5) | 9 mg/kg (N=4) | 450 mg q12h  (N=6) |
| Any TEAE | 5 (35.7)/7 | 0 | 1 (16.7)/1 | 1 (16.7)/4 | 4 (66.7)/10 | 5 (100)/17 | 3 (75.0)/9 | 4 (66.7)/5 |
| Mild | 4 (28.6)/5 | 0 | 1 (16.7)/1 | 1 (16.7)/4 | 3 (50.0)/9 | 5 (100)/15 | 3 (75.0)/7 | 4 (66.7)/4 |
| Moderate | 2 (14.3)/2 | 0 | 0 | 0 | 1 (16.7)/1 | 1 (20.0)/1 | 1 (25.0)/2 | 1 (16.7)/1 |
| Severe | 0 | 0 | 0 | 0 | 0 | 1 (20.0)/1 | 0 | 0 |
| Drug-related TEAE | 3 (21.4)/3 | 0 | 1 (16.7)/1 | 1 (16.7)/1 | 3 (50.0)/8 | 5 (100)/15 | 3 (75.0)/9 | 1 (16.7)/1 |
| TEAE occurring in >3 subjects |  |  |  |  |  |  |  |  |
| ALT increased | 0 | 0 | 0 | 0 | 1 (16.7)/1 | 1 (20.0)/1 | 1 (25.0)/1 | 0 |
| Headache | 2 (14.3)/2 | 0 | 0 | 0 | 1 (16.7)/1 | 1 (20.0)/1 | 0 | 1 (16.7)/2 |
| Infusion site pain | 0 | 0 | 0 | 0 | 1 (16.7)/1 | 4 (80.0)/5 | 2 (50.0)/2 | 0 |
| Pain in extremity | 0 | 0 | 0 | 1 (16.7)/1 | 0 | 1 (20.0)/2 | 1 (25.0)/1 | 0 |

ALT = alanine aminotransferase; N = number of subjects; TEAE = treatment-emergent adverse event

Supplemental Table 5. Treatment-emergent adverse events (TEAEs) after multiple ascending doses of BV100 (safety population)

|  | Number (%) of Subjects/Events | | |
| --- | --- | --- | --- |
|  | Placebo  (N=4) | 300 mg q24h (N=6) | 300 mg q12h (N=6) |
| Any TEAE | 0 | 6 (100)/21 | 5 (83.3)/63 |
| Mild | 0 | 6 (100)/15 | 5 (83.3)/56 |
| Moderate | 0 | 3 (50.0)/6 | 2 (33.3)/4 |
| Severe | 0 | 0 | 1 (6.7)/3 |
| Drug-related TEAE | 0 | 5 (83.3)/17 | 5 (83.3)/58 |
| TEAE occurring in at least 3 subjects |  |  |  |
| Headache | 0 | 2 (33.3)/3 | 1 (16.7)/2 |
| Infusion site pain | 0 | 4 (66.7)/5 | 5 (83.3)/14 |
| Infusion site erythema | 0 | 0 | 5 (83.3)/8 |
| Infusion site induration | 0 | 0 | 3 (50.0)/4 |
| Infusion site swelling | 0 | 1 (16.7)/1 | 2 (33.3)/3 |
| Lymphocyte count decreased | 0 | 1 (16.7)/1 | 3 (50.0)/3 |

N = number of subjects; TEAE = treatment-emergent adverse event
